# m^6^A modification erased by ALKBH5 promotes tumor growth and metastasis via regulation of YAP/ZEB1 axis in NSCLC

**DOI:** 10.1016/j.gendis.2025.101768

**Published:** 2025-07-11

**Authors:** Dan Jin, Weihua Di, Rui Li, Shuang Shao, Jiwei Guo

**Affiliations:** a Medical Research Center, Binzhou Medical University Hospital, Binzhou, Shandong 256603, China; b Department of Pain, Binzhou Medical University Hospital, Binzhou, Shandong 256603, China; c Gastroenterology Department, Binzhou Medical University Hospital, Binzhou, Shandong 256603, China

Early detection and effective treatment, as well as prevention of recurrence and metastasis, are crucial for patients with non-small cell lung cancer (NSCLC).^1^ Recent studies have shown that alkylation repair homolog 5 (ALKBH5) reverses m^6^A RNA methylation. Silencing ALKBH5 affects tumorigenesis and cancer progression under the action of m^6^A reading proteins, such as YTH domain family 3 (YTHDF3).^2^ The Yes-associated protein (YAP) pathway regulates cell proliferation, apoptosis, invasion, migration, and epithelial–mesenchymal transition (EMT),^3^ all processes that play a key role in tumor growth and metastasis.^4^ One notable EMT-related protein is zinc finger E-box–binding homeobox 1 (ZEB1), implicated in tumor progression.^5^ Currently, few studies have investigated the functions of these tumorigenic proteins in NSCLC. Here, our research revealed that ALKBH5, YTHDF3, YAP, and ZEB1 constitute the cellular axis regulating NSCLC cell proliferation, migration, invasion, and EMT in an m^6^A-dependent manner. Methylation inhibitor cycloleucine blocked this axis. Based on our findings, we propose that ALKBH5 plays an important supportive role in NSCLC tumor growth and metastasis. Thus, ALKBH5-mediated inhibition of *YAP* m^6^A modification is a promising novel target for NSCLC therapy.

To investigate the roles of ALKBH5 and YAP in NSCLC progression, we first analyzed their expression using The Cancer Genome Atlas (TCGA). The results showed that ALKBH5 was down-regulated in tumor tissues compared with levels in matched normal tissues (Fig. S1A), while YAP was up-regulated (Fig. S1B). In addition, shALKBH5 (knockdown using shRNA) and YAP overexpression (Fig. S1C) exerted similar promoting effects on regulating NSCLC cell proliferation (Fig. S1D and S1E), clone formation (Fig. S1F and S1G), migration (Fig. S1H and S1I), and EMT (Fig. S1J and S1K). These findings demonstrate that shALKBH5 and YAP play analogous roles in regulating NSCLC occurrence and development, with evidence indicating that shALKBH5 likely mediates these oncogenic effects by modulating YAP expression.

Given that ALKBH5 is an m^6^A demethylase, we next explored whether ALKBH5 regulated YAP in an m^6^A-dependent manner. ALKBH5 interacted with *YAP* mRNA (Fig. 1A), and m^6^A modification of *YAP* mRNA dose-dependently decreased in ALKBH5-ovexpressing A549 cells (Fig. 1B). Moreover, mRNA expression of *YAP* and target genes cysteine-rich 61 (*Cyr61*) and connective tissue growth factor (*CTGF*) were lower in A549 cells transfected with wild-type ALKBH5 than in cells transfected with ALKBH5 KD (H204A, a dominant catalytic variant) or the control plasmid (Fig. 1C). Furthermore, ALKBH5-mediated inhibitory effects of cellular growth (Fig. 1D; Fig. S2A), migration (Fig. S2B), invasion (Fig. S2C), and EMT (Fig. S2D) were mechanistically dependent on YAP expression. Next, we investigated whether m^6^A reader YTHDF3 played a role in the interaction between ALKBH5 and *YAP* mRNA. The RNA immunoprecipitation assay showed that YTHDF3 bound to *YAP* mRNA (Fig. 1E). When ALKBH5 inhibited m^6^A modification, we observed a significant decrease in YTHDF3 recognition of *YAP* mRNA via m^6^A (Fig. 1F). These data indicate that YTHDF3 exhibits specific binding affinity toward m^6^A-modified *YAP* mRNA.

**Figure 1:**
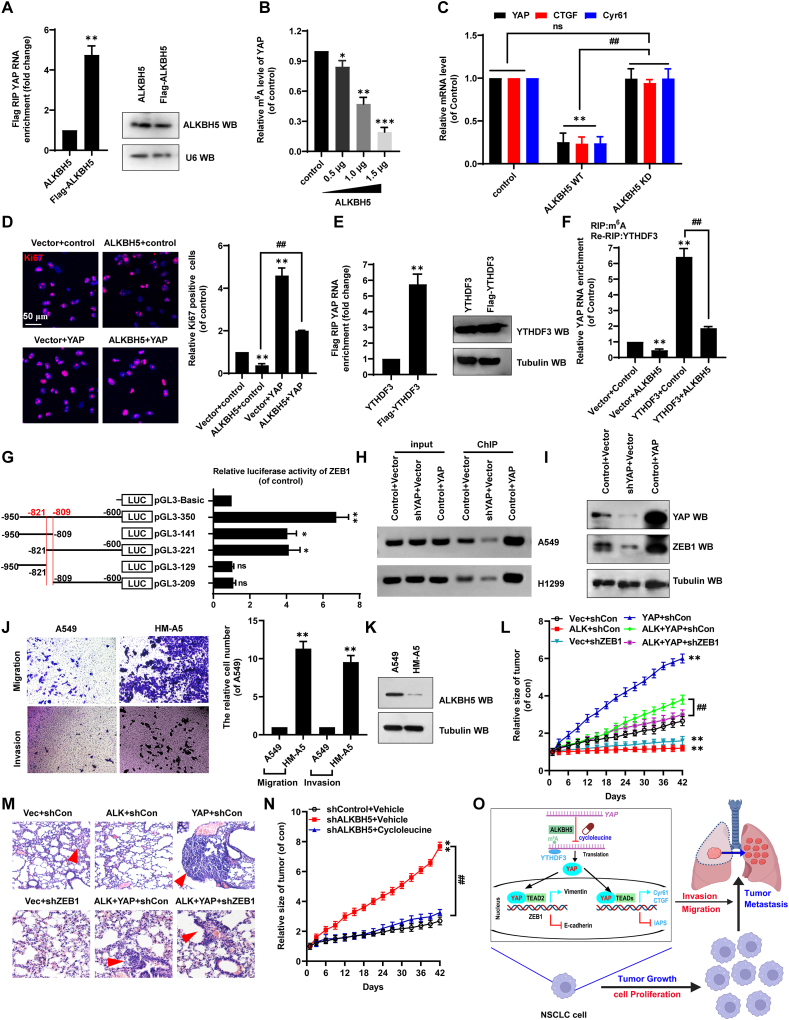
m^6^A promotes tumor growth and metastasis via regulation of YAP/ZEB1 axis in non-small cell lung cancer. **(A)** The interaction between ALKBH5 and *YAP* mRNA was determined by RIP assay. **(B)** The relative m^6^A modification within *YAP* mRNA was analyzed by m^6^A-RIP-quantitative PCR assay. **(C)** The mRNA levels of *YAP*, *CTGF*, and *Cyr61* were analyzed by quantitative PCR. **(D)** Immunofluorescence staining identified Ki-67-positive cells (Ki-67, a marker of cell proliferation). **(E)** The interaction between YTHDF3 and *YAP* mRNA was determined by RIP assay. **(F)** The relationship between YTHDF3 and *YAP* mRNA in the A549 cells with transfection of the indicated genes was detected by RIP assay. **(G)** The activities of different fragments of *ZEB1* promoter were detected by luciferase reporter gene assay. **(H)** The relation between YAP and *ZEB1* promoter was analyzed by quantitative chromatin immunoprecipitation. **(I)** The protein levels of YAP and ZEB1 were detected by western blotting. **(J)** The transwell assay of the cellular invasion and migration in A549 and HM-A5 cells. **(K)** The protein level of ALKBH5 in A549 and HM-A5 cells was determined by western blotting. **(L)** The tumor growth was detected in mice bearing xenografted A549 stable cells with the relevant genes. **(M)** Representative hematoxylin-eosin-stained microscopic images of metastatic lung tumors in the xenografted mouse (*n* = 5). **(N)** The tumor growth was detected in cycloleucine-treated xenografted mice. **(O)** The diagram of m^6^A promotes tumor growth and metastasis through the ALKBH5-YTHDF3-YAP-ZEB1 axis in non-small cell lung cancer. Results were presented as mean ± standard deviation of three independent experiments. ∗*p* < 0.05, ∗∗*p* < 0.01, ∗∗∗*p* < 0.001, or ^##^*p* < 0.01 indicates a significant difference between the indicated groups, and “ns” indicates no significance. YAP, Yes-associated protein; ZEB1, zinc finger E-box–binding homeobox 1; ALKBH5, alkylation repair homolog 5; Cyr61, cysteine-rich 61; CTGF, connective tissue growth factor; YTHDF3, YTH domain family 3; RIP, RNA immunoprecipitation.

Bioinformatics analysis then revealed that YAP interacted with ZEB1 (Fig. S3A). Additionally, JASPAR analysis showed that transcriptional enhanced associate domain 2 (TEAD2, a YAP transcription factor) preferentially bound to a universal consensus motif (Fig. S3B) inside the *ZEB1* promoter (from −821 to −809; Fig. S3C). The consensus motif was in pGL3-350, where luciferase activity was highest, suggesting that the predicted region (from −821 to −809) is the core *ZEB1* promoter site for interaction with YAP/TEAD2 (Fig. 1G; Fig. S3D). Moreover, co-transfecting NSCLC cells with YAP promoted interactions between YAP/TEAD2 and the *ZEB1* promoter (Fig. 1H), up-regulating ZEB1 (Fig. 1I); shYAP reduced those interactions and thus ZEB1 level from control conditions. Of note, we established highly metastatic A549-derived cells (HM-A5) through serial *in vivo* passage using xenograft models (Fig. S4A), and then investigated the roles of ALKBH5, YAP, and ZEB1 in this system. First, HM-A5 cells acquired enhanced metastatic capacity in a passage-dependent manner, likely due to stable epigenetic remodeling, which was consistently higher than that of the parental A549 line beyond passage 5 (Fig. S4A and S4B). Second, HM-A5 cells showed greater cellular invasion and migration than A549 cells (Fig. 1J). Third, ALKBH5 levels decreased (Fig. 1K; Fig. S4C) in HM-A5 cells (compared with levels in A549 cells), whereas YAP (Fig. S4D) and ZEB1 (Fig. S4E) levels increased. Collectively, ALKBH5-mediated m^6^A demethylation abrogated YTHDF3 recognition, thereby attenuating YAP-dependent ZEB1 transcriptional activation and concomitantly inhibiting proliferation, migration, invasion, and EMT in NSCLC cells.

To validate the *in vitro* results, we generated separate A549 cell lines stably expressing ALKBH5, YAP, or ZEB1 to explore their functions in tumor growth and metastasis *in vivo* (Fig. S5A). After model mice were subcutaneously implanted with various cell lines, the YAP group exhibited larger tumors (Fig. S5B) and faster tumor growth (Fig. 1L) than the vector group, whereas the opposite occurred in the ALKBH5 and shZEB1 groups. The ALKBH5+YAP group again exhibited larger tumors and faster tumor growth, in contrast with the ALKBH5 group. However, the ALKBH5+YAP + shZEB1 group had similar outcomes as the ALKBH5 group, with smaller tumors and slower growth (Fig. 1L). Overall survival of mice was negatively correlated with tumor growth (Fig. S5C). Moreover, quantitative PCR on xenograft tumor samples revealed that CTGF (Fig. S5D), ZEB1 (Fig. S5E), and vimentin (Fig. S5F) expression exhibited the same pattern as tumor growth, but E-cadherin expression had the opposite effect (Fig. S5G). The YAP group also produced larger and more metastatic lung cancer tumors than the vector group, while the ALKBH5 and shZEB1 groups had fewer and smaller metastatic tumors (Fig. 1M; Fig. S5H). Furthermore, compared with vehicle treatment (Fig. 1N), tumor growth and weight were inhibited in shALKBH5 groups treated with cycloleucine, an inhibitor of m^6^A modification on *YAP* mRNA (Fig. S5I and S5J). Overall, our *in vivo* experiments demonstrated that inhibiting shALKBH5-mediated m^6^A modification inhibited tumor growth and metastasis via regulating the YAP-ZEB1 axis (Fig. 1O).

In conclusion, this study identified a negative correlation between ALKBH5 and YAP expression, an interaction that contributes to regulating NSCLC tumor growth and metastasis. ALKBH5 inhibits m^6^A modification of *YAP* mRNA, thus limiting YTHDF3 action on the methylated site and regulating YAP expression. When activated, YAP increases ZEB1 expression to promote cellular EMT. As such, when cycloleucine blocked m^6^A modification of *YAP* mRNA, NSCLC tumor progression and metastasis were inhibited. Therefore, targeting *YAP* mRNA methylation may be a promising treatment strategy for NSCLC.

## CRediT authorship contribution statement

**Dan Jin:** Validation, Methodology, Supervision, Investigation. **Weihua Di:** Writing – review & editing. **Rui Li:** Writing – original draft, Software, Data curation, Writing – review & editing, Supervision, Resources. **Shuang Shao:** Software, Formal analysis, Supervision, Investigation. **Jiwei Guo:** Writing – original draft, Conceptualization, Writing – review & editing, Supervision.

## Ethics declaration

The animal protocols were reviewed and approved by the Ethics Committee of Binzhou Medical University Hospital, Shandong, China (No. 2018-019-04).

## Funding

This work was supported by the “Youth Innovation Science and Technology Plan” of Colleges and Universities in Shandong Province, China (2020KJK002), Qilu Health Project (Shandong, China), and BoHai Contribution Expert (Shandong, China).

## Conflict of interests

The authors declared no conflict of interests.
