# Supplementary material for: m6A modification erased by ALKBH5 promotes tumor growth and metastasis via regulation of YAP/ZEB1 axis in NSCLC
Source: Genes Dis. 2025 Jul 11;13(2):101768. doi: 10.1016/j.gendis.2025.101768 (PMC12596588; doi:10.1016/j.gendis.2025.101768)
Supplement: Multimedia component 1 [file mmc1.docx]

**m^6^A modification erased by ALKBH5 promotes tumor growth and metastasis via regulation of YAP/ZEB1 axis in the NSCLC**

**Dan Jin^a^, Weihua Di^b^, Rui Li^a, c^, Shuang Shao^a^, Jiwei Guo^a, c⁎^**

^a^ Medical Research Center, Binzhou Medical University Hospital, Binzhou, Shandong, 256603, China

^b^ Department of Pain, Binzhou Medical University Hospital, Binzhou, Shandong, 256603, China

^c^ Gastroenterology Department, Binzhou Medical University Hospital, Binzhou, Shandong, 256603, China

^*^ Corresponding author. Medical Research Center, Binzhou Medical University Hospital, Binzhou, Shandong, 256603, China.

E-mail address: gjw0510@mail.nankai.edu.cn (J. Guo).

**Materials and Methods**

**Molecular biology**

The recombinant genes YAP, YTHDF3, ALKBH5 and ZEB1 were structured with the pcDNA 3.1 vector (Invitrogen, Carlsbad, CA, USA). The Flag (DYKDDDDK) epitope tag was added by PCR.

**Cell lines and culture**

NSCLC cell lines were purchased from American Type Culture Collections (Manassas, VA). Cell lines were cultivated in RPMI-1640 medium (Biological Industries, Beit Haemek, Israel) supplemented with 10% FBS (Hyclone, USA), penicillin/streptomycin (100 mg/mL). Culture flasks were kept at 37 ˚C in a humid incubator with 5% CO_2_.

**Over-expression and knockdown of genes**

The indicated genes with shRNA (1.5 μg) and overexpressing plasmid (2 μg) were transfected into NSCLC cells using Lipofectamine 3000 (Invitrogen, Carlsbad, CA) for knockdown and over-expression of target genes, followed by analysis 48 h later. The target sequences for knockdown of the indicated genes are shown in Table S1.

**RNA isolation and** **reverse transcription (RT)-qPCR assay**

The total RNA of NSCLC cells was isolated by TRIzol reagent (TransGen Biotech, Beijing, China) and reversely transcribed into first-strand cDNA using a TransScript All-in-One First-Strand cDNA Synthesis Kit (TransGen Biotech). cDNAs were used in RT-qPCR assay with the human *GAPDH* gene as an internal control. The final RT-qPCR reaction mix contained 10 μL Bestar^®^ SYBR Green qPCR MasterMix. Amplification was performed as follows: a denaturation step at 94 °C for 5 min, followed by 35 cycles of amplification at 94 °C for 30 s, 58 °C for 30 s and 72 °C for 30 s. The reaction was stopped at 25°C for 5 min. The relative expression levels were detected and analyzed by CFX96™ Real-Time PCR System (1855195, Bio-Rad) based on the formula of 2^-ΔΔct^. Gene expression was normalized to that of *GAPDH* in the quantification analysis. All primer sequences used in this study are shown in Table S2.

**Western blot analysis**

Cells were transfection with the relevant plasmids and cultured for 48 h. For WB analysis, cells were lysed in NP-40 buffer (10 mM Tris pH 7.4, 1% Triton X-100, 150 mM NaCl, 1 mM PMSF, 1 mM EDTA pH 8.0, 1 mM EGTA pH 8.0, and 0.5% NP-40) at 25 ˚C for 20 min. After centrifuging at 12,000 rpm for 15 min, the supernatant was harvested, and the protein concentration was determined using a BCA Kit (Solarbio, PC0020, Beijing, China) following manufacturer instructions. Proteins were separated by sodium dodecyl sulfate–polyacrylamide gel electrophoresis (BOSTER, AR0047, Wuhan, China) and electroblotted on polyvinylidene difluoride membrane (ISEQ00010, Millipore, Burlington, MA, USA). Membranes were incubated with primary antibody (1:1000 dilution) at 4 °C overnight, then incubated with the secondary antibody (1:2000 dilution) for 2 h at 25 °C. Enhanced chemiluminescence signals (UElandy, S6009M, Suzhou, China) were detected using the Image Lab software. The first antibodies used in our study were 1:1000 rabbit anti-Flag (sc-166384, Santa Cruz, Dallas, TX, USA) and 1:1000 Abcam (Cambridge, UK) antibody of YAP (ab56701), ALKBH5 (ab234528), YTHDF3 (ab220161), ZEB1 (ab303480), E-cadherin (ab219332), Vimentin (ab45939), and Tubulin (ab6046). Secondary antibodies HRP-linked goat antirabbit IgG (H+L; 511203) were obtained from ZENBIO. Molecular imager (ChemiDocXRS+, Bio-Rad, Hercules, CA, USA) was used to get the result and quantified using Image J software.

**Immunofluorescence staining**

Cells were seeded in 24-well plates at 5x10^4^ per well. After cellular transfection 48 h, cells were fixed as previously described [1]. Cells were incubated with primary antibodies Ki67 (ab15580, Abcam) overnight at 4°C. As a negative control, slides incubated with BSA were stained in parallel. Then cells were incubated with the secondary antibody Alexa Fluor 594 AffiniPure Goat Anti-Rabbit IgG (H+L; 33112ES60, Yeasen Biotechnology Co., Ltd., Shanghai, China) diluted with BSA for 2 h at 25 °C. Cells were counterstained with 4',6-diamidino 2-phenylindole (DAPI) and visualized with an advanced fluorescence microscope (Olympus BX63) and quantified using ImageJ software.

**CCK8 assays**

Cells were seeded into a 96-well plate at a density of 3000 cells per well. Cell growth and viability were analyzed by CCK8 assays in 96-well plates. NSCLC cells were transfected of the indicated genes for 36 h and then incubated with CCK8 for 2 h at 37 °C. The absorption and reference wavelengths were measured using a BioTek Synergy H1 hybrid microplate reader at 450 and 630 nm, respectively.

**Wound healing assay**

Cells were seeded into 12-well plate at a density of 1 × 10^5^ cells per well and transfected of the indicated genes for 36 h. When cells reached 80% confluence, a gap was created using a 200 µL tip of a pipette and washed with phosphate-buffered saline (PBS). Cells were incubated with serum-free medium for 36 h before photomicrographs were taken with the IX53 inverted microscope and DP73 color camera (Olympus). Relative distance of the remaining wound length was calculated by Image J (National Institutes of Health).

**Colony formation assay**

Cells were seeded into a six-well plate at a density of 700 cells per well and cultured for 14 d in RPMI1640 medium. Thereafter, cells were fixed with 4% paraformaldehyde (A500684, Sangon Biotech) and stained with crystal violet (A100528, Sangon Biotech) for 15 min. Molecular imager (ChemiDocXRS+, Bio-Rad, Hercules, CA, USA) was used to screen and analyze the densitometry of each visual field using the ImageJ software and quantified using ImageJ software.

**Transwell assay**

The transwell assays for cellular migration and invasion growth were performed as previously described [1]. In the migration assay, 5 × 10^3^ cells were suspended in 200 μL of RPMI 1640 medium (Biological Industries, Beit Haemek, Israel) without FBS and seeded on the top chamber of transwell inserts (3422, Corning). The lower chambers contained RPMI1640 medium with 10% FBS. After incubation for 48 h, the chamber was rinsed three times with PBS, then dyed with crystal violet for 30 s and rinsed again with PBS five times to obtain a transparent background. For the invasion assay, the upper surface of the transwell filter membrane (3422, Corning) was pre-coated with a 1:8 dilution of Matrigel^®^ matrix (354234, Corning) in serum-free medium using the chilled pipette tips prior to cell inoculation. All other processes were the same as for the transwell migration assay. Plates were imaged using the IX53 inverted fluorescence microscope and the DP73 color camera (Olympus) and quantified using ImageJ software.

**RNA immunoprecipitation assay**

All cell precipitates were chilled in polysome lysis buffer for 10 min on ice. All lysates were centrifuged at 13,000 g for 10 min; some supernatant was preserved as an input sample, and the rest was treated with antibody-rProtein A/G MagPoly Beads (SM015005, Changzhou Smart-Lifesciences Biotechnology Co., Ltd., Changzhou, China) mixture, which was preincubated at 25℃ for 30 min and vortexed at 4 °C overnight. Samples were washed six times with NT-2 buffer and further treated with proteinase K buffer (MedChemExpress; HY-108717). After heating in a water bath at 55 °C for 30 min, the RNA was extracted at 5,000 g for 15 s using a mini centrifuge (D1008E, DLAB Scientific, Beijing, China) and by collecting the supernatant of the NT-2 buffer. The antibodies used in the above experiments are those against Flag (ab205606, Abcam), m^6^A (ab208577, Abcam) and YTHDF3 (ab220161, Abcam) that were used for RNA immunoprecipitation assay.

**ChIP**

This experiment was conducted according to the instructions of the ChIP-IT High Sensitivity Kit (53040, Active Motif, Carlsbad, CA, USA). A primary antibody specific

to YAP (ab52771, Abcam) were used for chromatin immunoprecipitation. And primer sequences for the enhancer of ZEB1 are listed in Table S3.

**MS2 coat protein system to enrich mRNA**

The MS2 system was used as described previously [2]. Briefly, A549 with stable expressing YAP-MS2 (YAP-MS2) were co-transfected with MS2-GFP and relevant genes. The transfected cells were lysed immunoprecipitated by GFP antibody to enrich *YAP* mRNA then performed the following experiment.

**m^6^A mRNA immunoprecipitation**

The total m^6^A content of total *YAP* mRNA was determined using an m^6^A methylation quantification kit (P-9008-96, EpiGentek, USA). Briefly, after total RNA was isolated and purified, the bind RNA was planted to the assay wells and cultured with the capture antibody. After that, the wells were washed, and the detection antibody and enhancer solution were added. The m^6^A level was detected according to the fluorescence after the wells were incubated with the fluoro developer solution.

**qPCR for** **m^6^A-RIP**

Reverse transcription was performed on 10 μL m^6^A PolyA+ RNA from the MeRIP with the iScript cDNA synthesis kit (Bio-Rad Laboratories, Hercules, CA). After diluting cDNA two-fold, quantitative real-time PCR was performed using the CFX96™ Real-Time PCR System (1855195, Bio-Rad) and primers from Integrated DNA Technologies, Inc. (Coralville, Iowa). Primer efficiency was verified to be over 95% for all primer sets used. Quantification of mRNA from the m^6^A-RIP was carried out via 2^-ΔΔCT^ analysis against non-immunoprecipitated input RNA. All real-time PCR primer sets were designed so the products would span at least one intron (> 1 kb when possible), and amplification of a single product was confirmed by agarose gel visualization and/or melting curve analysis. Primers for m^6^A-RIP are listed in Table S4.

**luciferase reporter assay**

To construct the core region of *ZEB1* promoter, the region of *ZEB1* was amplified by PCR from the human cDNA of A549 cells and were inserted into the upstream of the pGL3-Basic vector (Promega, Madison, WI, USA) via KpnI and XhoI sites to generate ZEB1luc. Thereafter, we use the Firefly Luciferase Reporter Gene Assay Kit (#RG005, Beyotime, China) to detect the promoter activities. The PGL3-basic plasmid was used as a negative control. Data was normalized against Renilla luciferase activity.

**Analysis of publicly available datasets**

The correlation between YAP and ALKBH5 level and prognostic outcome of patients were used Kaplan-Meier survival curves in NSCLC patients (www.kmplot.com/analysis and <https://www.cancer.gov/>) [3].

**A549** **stable cell lines**

A549 cells were seeded into 6-well plates. After 24 h, the relevant plasmids using pcDNA 3.1 as a vector were transfected into A549 cells using Lipofectamine 3000 (Invitrogen, Carlsbad, CA) according to the manufacturer's protocol, when the cellular confluency reached 80%. After 48 h, the transfected cells were seeded at a 1:5 ratio and switched to a selection medium containing G418 (400 mg/mL). The G418 concentration was increased by 100 mg/mL every other day until reaching a maintenance concentration of 800 mg/mL, and then these transfected A549 cells were sustained for 3 weeks to form at a monoclonal colon. Ten clones were selected for amplification culture and intermittently treated with 400 mg/mL G418. During this period, qPCR was performed weekly to monitor the expression of relevant genes. A549 stable cell lines expressing the relevant desired genes were ultimately obtained through this screening process.

**Immunohistochemical analysis**

Hematoxylin and eosin and immunohistochemistry staining of paraffin sections were performed as previously reported [4]. Briefly, images were observed via bright field microscopy (BX63, Olympus) and quantified using Image J software. For immunofluorescence staining, tissues were immersed in 30% sucrose solution overnight and embedded in Tissue Tek OCT compound (4583, Sakura Finetek, Tokyo, Japan). Subsequently, frozen sections (5 μM) were prepared using a cryostat (Leica CM1950, Leica, Wetzlar, Germany). Frozen sections were incubated with 5% normal goat serum (Zhongshan Golden Bridge Biotechnology, Beijing, China) for 40 min at 37 °C. Primary antibodies against CTGF (ab6992, Abcam), ZEB1 (ab303480, Abcam), Vimentin (ab137321, Abcam) and E-cadherin (ab314063, Abcam) were diluted 1:100 in PBS, applied to cover sections, and incubated at 4 °C for 12 h. Sections incubated with only PBS were used as negative controls. Sections were then incubated with specific HRP/DAB Detection IHC Kit (ab64261, Abcam). DAPI was used to co-stain the cell nuclei. Images were acquired under an advanced fluorescence microscope (BX63, Olympus BX63) and quantified using Image J software.

**In vivo experiments**

Four-week-old BALB/c nude mice (female, 15-18 g) were purchased from Ji'nan Pengyue Laboratory Animal Breeding Co., Ltd. (Jinan, China) and kept in a specific pathogen-free laboratory animal room. The use of animals was approved by the Animal Experiments Ethical Committee of Binzhou Medical University Hospital and performed according to the Guide for Care and Use of Laboratory Animals. To establish highly metastatic HM-A5 cells, tumor-bearing mice were generated by subcutaneous injection of 5×10⁶ A549 stable cells. After 5 weeks, the mice were euthanized, and lung metastatic tumor cells were isolated, digested, and cultured (designated as HM-A1). These HM-A1 cells were then subcutaneously injected into BALB/c nude mice （5×10⁶ cells per mouse）. Following 5 weeks, the mice were euthanized, and lung metastatic tumor cells were collected, cultured, and designated as HM-A2. HM-A3, HM-A4, and HM-A5 cells were sequentially obtained by repeating this procedure. For analysis of the tumor metastasis of ALKBH5, YAP and ZEB1 *in vivo*, a tumor bearing mouse model was developed via subcutaneous injection of the indicated 5 × 10^6^ lung cancer cells with stable relevant plasmids and randomly divided into indicated groups (five mice per group). When tumors reached approximately 5 mm in diameter, the tumor size and body weight of mice were measured every alternate day. Tumor volume was estimated as 0.5 × a^2^ × b (where a and b represent a tumors short and long diameter, respectively). All mice were euthanized after 7 weeks and tumors were excised for further analysis.

**Ethics approval**

The experimental protocol was approved by the Research Ethics Committee of Binzhou Medical University, China (No. 2018–019-04 for mice experiments *in vivo*).

**Statistical analysis**

Data are presented as mean ± standard error of mean (SEM). Graphpad Prism Software 9.0 (San Diego, CA, USA) was used for statistical analysis. Comparisons of two groups were analyzed with two-tailed Student’s *t*-test. Multiple comparisons were performed using one-way analysis of variance (ANOVA) or two-way ANOVA. Differences were considered significant at *p* < 0.05.

**References**

[1] Guo J, Wu Y, Du J, et, al. Deregulation of UBE2C-mediated autophagy repression aggravates NSCLC progression. Oncogenesis. 2018;7(6):49.

[2] Heinrich S, Sidler CL, Azzalin CM, et, al. Stem-loop RNA labeling can affect nuclear and cytoplasmic mRNA processing. RNA. 2017;23(2):134-141.

[3] Gyorffy B, Lanczky A, Eklund AC, et, al. An online survival analysis tool to rapidly assess the effect of 22,277 genes on breast cancer prognosis using microarray data of 1,809 patients. Breast Cancer Res Treat. 2010;123(3):725-731.

[4] Wang F, Zhang S, Sun F, et, al. Anti-angiogenesis and anti-immunosuppression gene therapy through targeting COUP-TFII in an in situ glioblastoma mouse model. Cancer Gene Ther. 2024;31(8):1135-1150. 

**Supplementary Data**

**Table S1 Sequences for knockdown.**

| **Genes** | **Target** **Sequences** | |
| --- | --- | --- |
| *shYAP* | AGGTGATACTATCAACCAA | |
| *shZeb1* | GGTAGATGGTAATGTAATAT  CCTCATAGTCGCTGCGCTCG  TTCTCCGAACGTGTCACGA |  |
| *shAlkbh5* |  |  |
| *shcontrol* |  |  |
|  |  |  |

| **Genes** | **Forward** | **Reverse** |
| --- | --- | --- |
| *Alkbh5* | GCCTATTCGGGTGTCGGAAC | CTGAGGCCGTATGCAGTGAG |
| *Yap* | GGACCCCAGACGACTTCCTC | CCTTCCAGTGTGCCAAGGTC |
| *Cyr61* | GGTCAAAGTTACCGGGCAGT | GGAGGCATCGAATCCCAGC |
| *Ctgf* | ACCGACTGGAAGACACGTTTG | CCAGGTCAGCTTCGCAAGG |
| *Zeb1* | CGAACCCGCGGCGCAATA | CCAGCAGTTCTTAGCATTCC |
| *E-cadherin* | ACCATTAACAGGAACACAGG | CAGTCACTTTCAGTGTGGTG |
| *Vimentin* | CGCCAACTACATCGACAAGG | CTGGTCCACCTGCCGGCGC |
| *Gapdh* | CTCCTCCTGTTCGACAGTCA | CCCAATACGACCAAATCCG |
| **Table S3 Primers for CHIP.** | | |

| **Genes** | **Forward** | **Reverse** |
| --- | --- | --- |
| *Zeb1* | CGGCGGAGTCAGGGGGAGC | GGTTTCCCCCCAAGCGAAC |
|  |  |  |

**Table S2 Primers for RT- qPCR.**

**Table S4 Primers for m^6^A-RIP.**

| **Genes** | **Forward** | **Reverse** |
| --- | --- | --- |
| *YAP* | TGCGCGTCGGGGGAGGCAGAAG | GGAATGAGCTCGAACATGCTG |

**
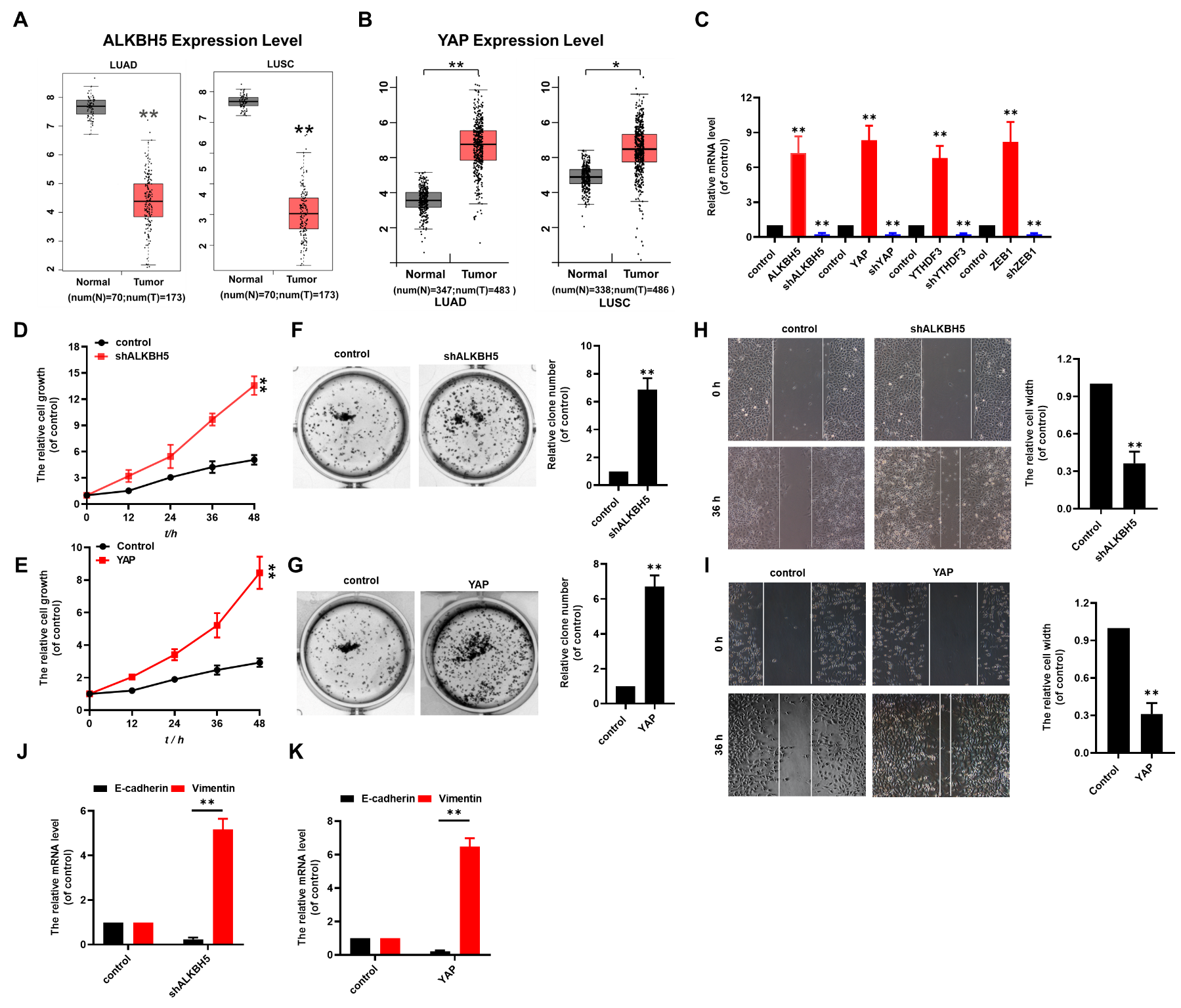
**

**Figure S1 Ectopic expression of YAP and ALKBH5 regulates cell proliferation, migration, and EMT in A549 cells.** (A) The ALKBH5 level was analyzed by TCGA database (***N****ormal*=70, ***T****umor*=173). (B) The TCGA database indicated that YAP was higher in tumor tissues than their normal tissues in the lung adenocarcinoma (LUAD, *N*=347, *T*=483) and lung squamous carcinoma (LUSC, *N*=338, *T*=486), respectively. (C-J) A549 cells were knockdown of ALKBH5 used shALKBH5 and overexpression of YAP used pcDNA3.1-YAP, respectively. (C) The mRNA levels of *ALKBH5* and *YAP* were detected by qPCR assay. (D, E) The cellular growth was detected by CCK8 assay. (F, G) The number of colons was detected by the colony formation assay. (H, I) The cellular migration growth was detected by the scratch assay. (J, K) The mRNA levels of *E-cadherin* and *Vimentin* were detected by qPCR assay. Results were presented as mean ± SD of three independent experiments. ^*^*P*<0.05 or ^**^*P*<0.01 indicates a significant difference between the indicated groups.

**
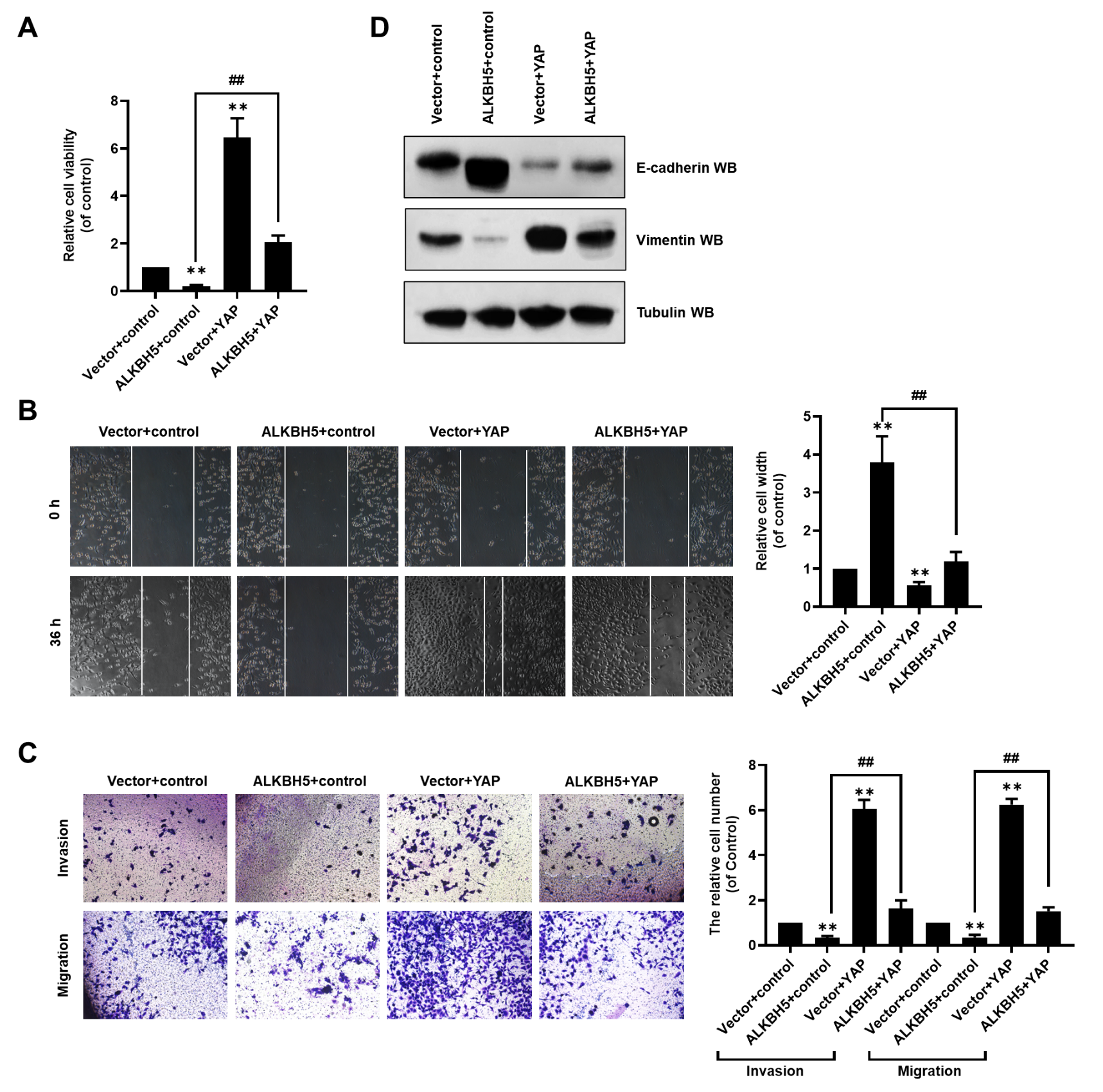
**

**Figure S2 ALKBH5 inhibits cellular growth and migration via regulation of YAP in A549 cells.** A549 cells were transfected by *control*, *ALKBH5*, *YAP* or co-transfected by *ALKBH5* and *YAP*, respectively. (A) The CCK8 assay analyzed the cellular viability. (B) The scratch assay analyzed the cellular migration growth. (C) The transwell assay detected cellular migration and invasion growth. (D) WB assay detected the E-cadherin and Vimentin levels. Results were presented as mean ± SD of three independent experiments. ^**^*P*<0.01 or ^##^*P*<0.01 indicates a significant difference between the indicated groups.

**
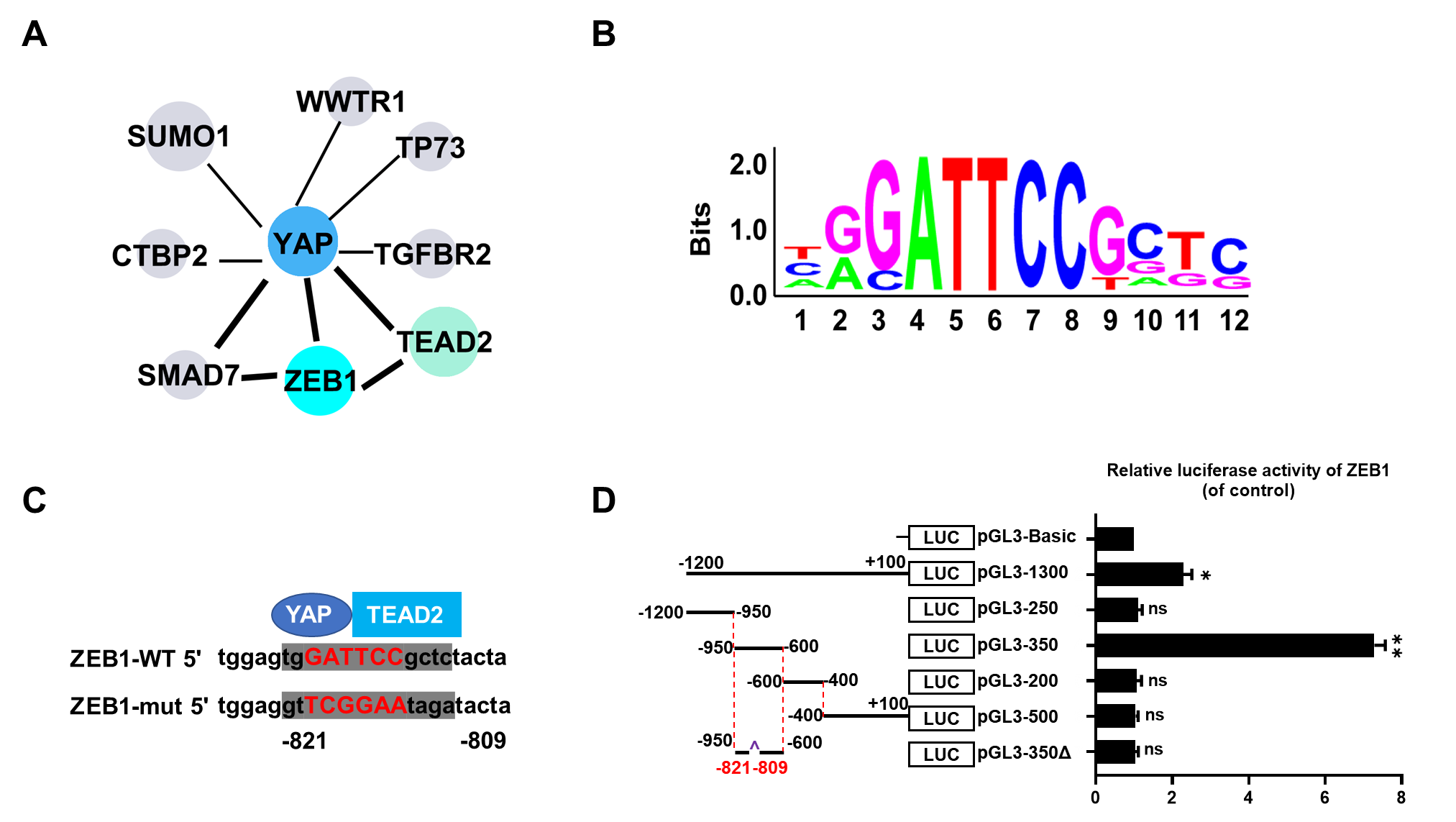
**

**Figure S3 YAP promotes the transcription of *ZEB1*.**

(A) Bioinformatics predicted the relation between YAP and ZEB1 (<https://thebiogrid.org/>). (B, C) The JASPAR database showed the conservative binding motif of TEAD2 (B) and the binding sites within the *ZEB1* promotor (C). (D) The luciferase reporter gene assays detected the activities of different fragments of *ZEB1* promoter in A549 cells with co-transfection with the *YAP*. Results were presented as mean ± SD of three independent experiments. ^*^*P*<0.05 or ^**^*P*<0.01 indicates a significant difference between the indicated groups. *ns*, not significant.

**
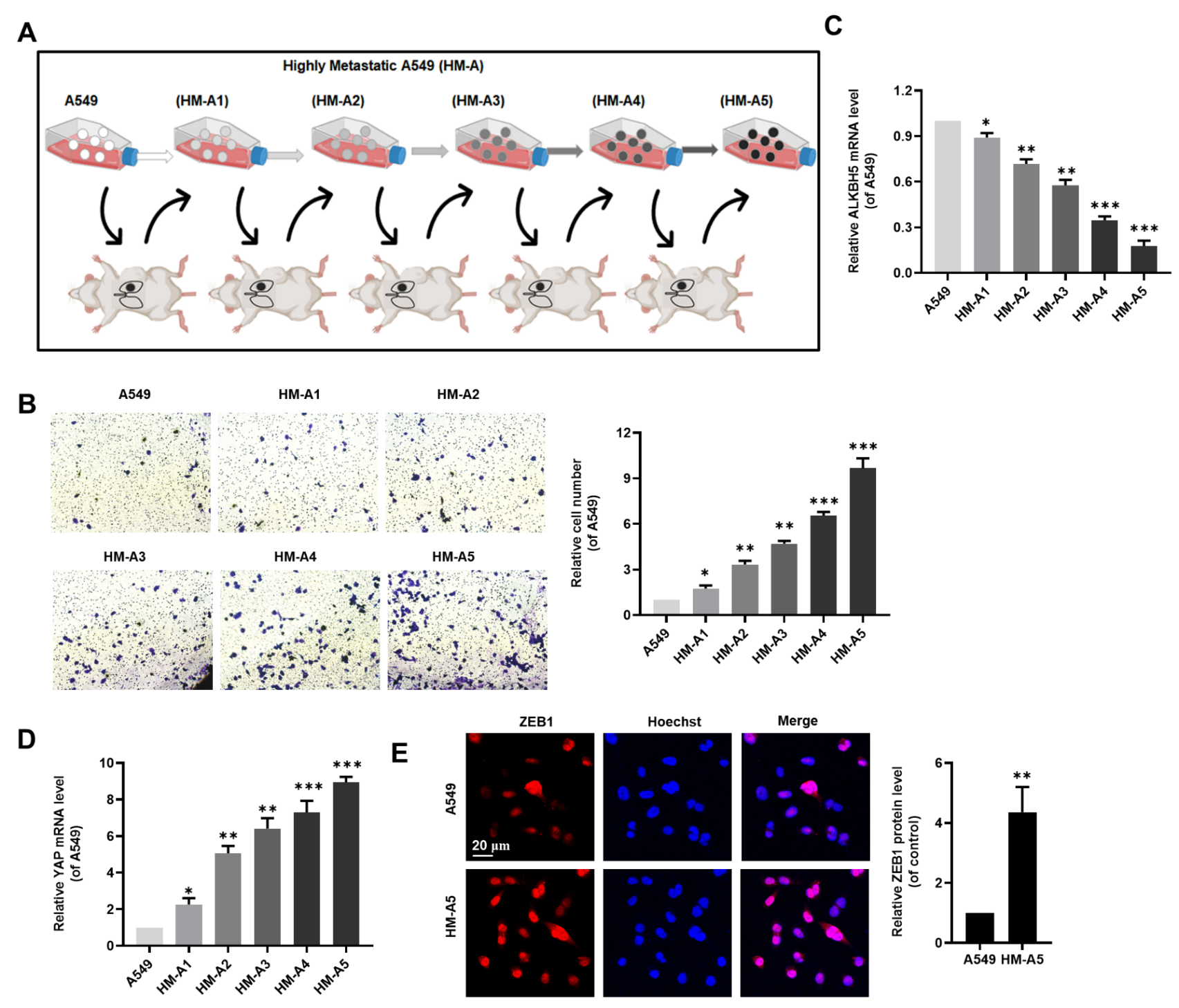
**

**Figure S4 ALKBH5 regulates cellular migration and invasion growth via regulation of YAP/ZEB1 axis.** (A) A schematic model of establishment for highly metastatic A549 (HM-A1 to HM-A5) using BALB/c athymic nude mice. (B) The metastatic capacity was detected by transwell assay in A549 and HM-A1 to HM-A5 cells, respectively. (C, D) The qPCR assay of the *ALKBH5* (C) and *YAP* (D) levels in A549 and HM-A1 to HM-A5 cells, respectively. (E) The ZEB1 levels in HM-A5 and A549 determined by the immunofluorescent assay. Results were presented as mean ± SD of three independent experiments. ^*^*P*<0.05, ^**^*P*<0.01 or ^***^*P*<0.001 indicates a significant difference between the indicated groups.

**
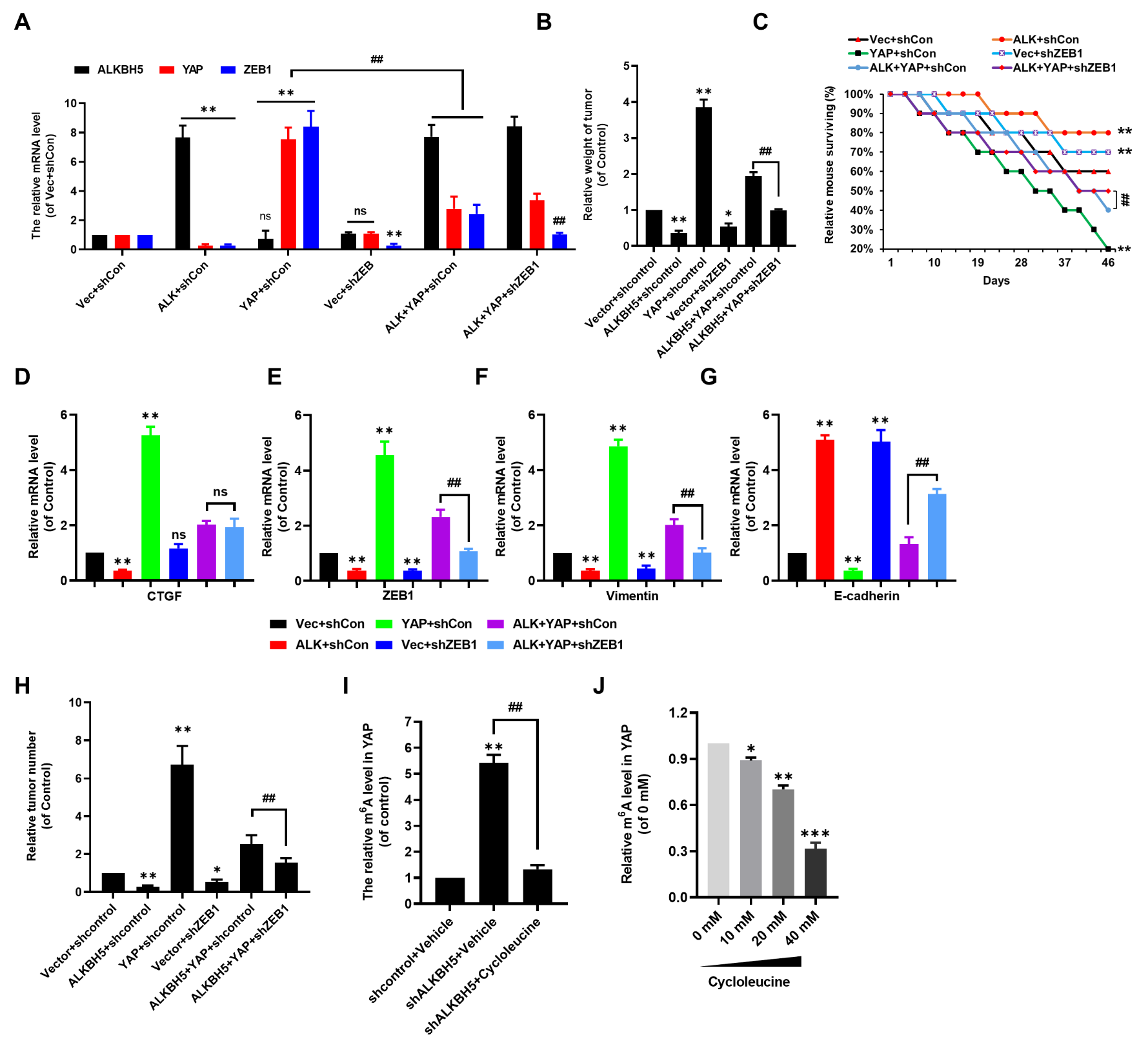
**

**Figure S5** **ALKBH5 restrains tumor growth via YAP-ZEB1 axis *in vivo*.** (A) The *ALKBH5*, *YAP* and *ZEB1* mRNA levels were detected in the A549 cell lines stably expressing the relevant genes. (B, C) The tumor weight (B) and overall survival (C) were detected in mice with the relevant xenografted A549 cells (*n*=10). (D-G) The mRNA levels of *CTGF* (D), *ZEB1* (E), *Vimentin* (F) and *E-cadherin* (G) were detected in tumors from mice with the relevant xenografted A549 cells determined by qPCR assay. (H) The number of lung tumors in A549-xenografted mice was quantified by immunohistochemistry. (I) A549 cells were transfected with shALKBH5 and then treated with Vehicle or 20 mM cycloleucine for 48h. The m^6^A within *YAP* mRNA was detected by m^6^A-RIP-qPCR. (J) A549 cells were treated with Vehicle or 10 mM, 20 mM or 40 mM cycloleucine for 48h, respectively. The m^6^A within *YAP* mRNA was detected by m^6^A-RIP-qPCR. Results were presented as mean ± SD of three independent experiments. ^*^*P*<0.05, ^**^*P*<0.01, ^***^*P*<0.001 or ^##^*P*<0.01 indicates a significant difference between the indicated groups.
